# Spin dynamics in superconductor/ferromagnetic insulator hybrid structures with precessing magnetization

**DOI:** 10.3762/bjnano.14.22

**Published:** 2023-02-21

**Authors:** Yaroslav V Turkin, Nataliya Pugach

**Affiliations:** 1 HSE University, Moscow 101000, Russia; 2 Vernadsky Crimean Federal University, Simferopol 295007

**Keywords:** ferromagnetic resonance, proximity effect, superconducting spintronics

## Abstract

The main goal of the present work is the description of the dynamics of spin current and induced magnetization inside a superconducting film S that is in contact with a ferromagnetic insulator layer FI. Spin current and induced magnetization are calculated not only at the interface of the S/FI hybrid structure, but also inside the superconducting film. The new and interesting predicted effect is the frequency dependence of the induced magnetization with a maximum appearing at high temperatures. It is also shown that the increase of the magnetization precession frequency can drastically change the spin distribution of quasiparticles at the S/FI interface.

## Introduction

Creation and manipulation of spin flows in superconducting hybrid systems have become a very active research area during the last decade because of the possibility to create spin supercurrents with much larger relaxation lengths and spin lifetimes [1]. The creation of persistent spin currents in superconductors opens new ways for the development of prospective spintronic devices such as magnon transistors [2–3] and superconducting magnon crystals [4]. In this context, the challenge of superconducting spin injection is one of the central problems in modern superconducting spintronics. There are several ways of spin current injection into a superconductor, for example, the spin Hall effect [5], the spin Seebek effect [6], and ferromagnetic resonance spin pumping [7–8]. The spin pumping technique in hybrid structures consisting of a ferromagnetic insulator and a superconductor is considered to be the most preferable way to inject spin currents because of the absence of Joule heating. Moreover, proximity coupling between magnetic excitations plays a crucial role in ferromagnetic Josephson junctions [9–12] and mesoscopic structures [13]. Recent experimental research [5,8,14] shows that the interaction between the superconducting correlations and spin waves influences the dynamics of both superconducting and magnetic films. Interfacial exchange interaction between Cooper pairs and magnons results in a nonstationary induced magnetization and spin currents in the superconducting film and changes the magnetic excitation spectrum inside the ferromagnetic insulator [15]. Despite the large number of discussions in experimental works, there is no clear understanding of the interplay between superconducting and magnetic excitations inside proximity-coupled hybrid structures. That is why developing a consistent theory of the inverse proximity effect is one of the central topics of modern superconducting spintronics. There is a series of theoretical papers [7,16–19] describing spin current injection and induced magnetization generation in microscopic [7,16] and quasiclassical [17–19] frameworks. However, the main subject of these works is the magnetic excitation spectrum in hybrid structures. Most of the works ignore the dynamics of nonuniform distributions of induced magnetization and spin current inside the superconducting film, which can be called the “dynamic inverse proximity effect”. Distributions of spin current and induced magnetization were calculated in recent works [20–21], where the authors investigate spin current flow through Josephson-like trilayer structures.

The proximity effect is the penetration of superconducting correlations in an adjacent nonsuperconducting layer, which serves as an origin of the Josephson effect, for example. While the reverse influence of a magnetic layer on a superconducting condensate is called the inverse proximity effect. Both spin current and induced magnetization in the superconductor originate from singlet–triplet Cooper pair conversion, which is the main mechanism of the inverse proximity effect. The magnetization in a superconductor is induced by the proximity in a stationary case, and the spin current is pumped only via magnetic dynamics in the adjacent layer. The quasiclassical theory of proximity effect in superconductor/ferromagnetic insulator hybrid structures was applied to describe nonstationary phenomena, such as generation of spin transfer torques, nonuniform thermoelectric effects, and domain wall movement. The theoretical description of the dynamic proximity effect is the more complex task because of the double time structure of the nonstationary Usadel equation. The recent successes in the theory of boundary conditions for quasiclassical approximations [22–23] enabled the development of adequate models of the proximity effect in different types of superconducting hybrid structures. Quasiclassical boundary conditions can successfully describe the interfaces between, among other things, a superconductor and weak or strong ferromagnets [22–24], normal metals [25–27], and half-metals [28]. The first attempts to implement nonstationary, adiabatic, quasiclassical boundary conditions were made in [18–19]. In these works, calculations based on Usadel equations combined with adiabatic, nonstationary boundary conditions were made. We show that the adiabatic approximation is useful in a wide range of magnetization precession frequencies. The main goal of our theory is to describe the dynamic perturbations produced by the spin current and induced magnetization inside the superconducting film in contact with a ferromagnetic insulator layer with precessing magnetization. Distributions of spin current and induced magnetization originating from the dynamic proximity effect in aluminium were recently calculated [29]. Another important problem that occurs during consideration of the dynamic inverse proximity effect is the nonequilibrium behavior of quasiparticles at the S/FI interface. In this work, we present our new results that prove that adiabatic dynamics of quasiparticles into the superconducting layer can be changed by spin pumping from the adjacent ferromagnetic insulating layer.

## Model

**Figure 1:**
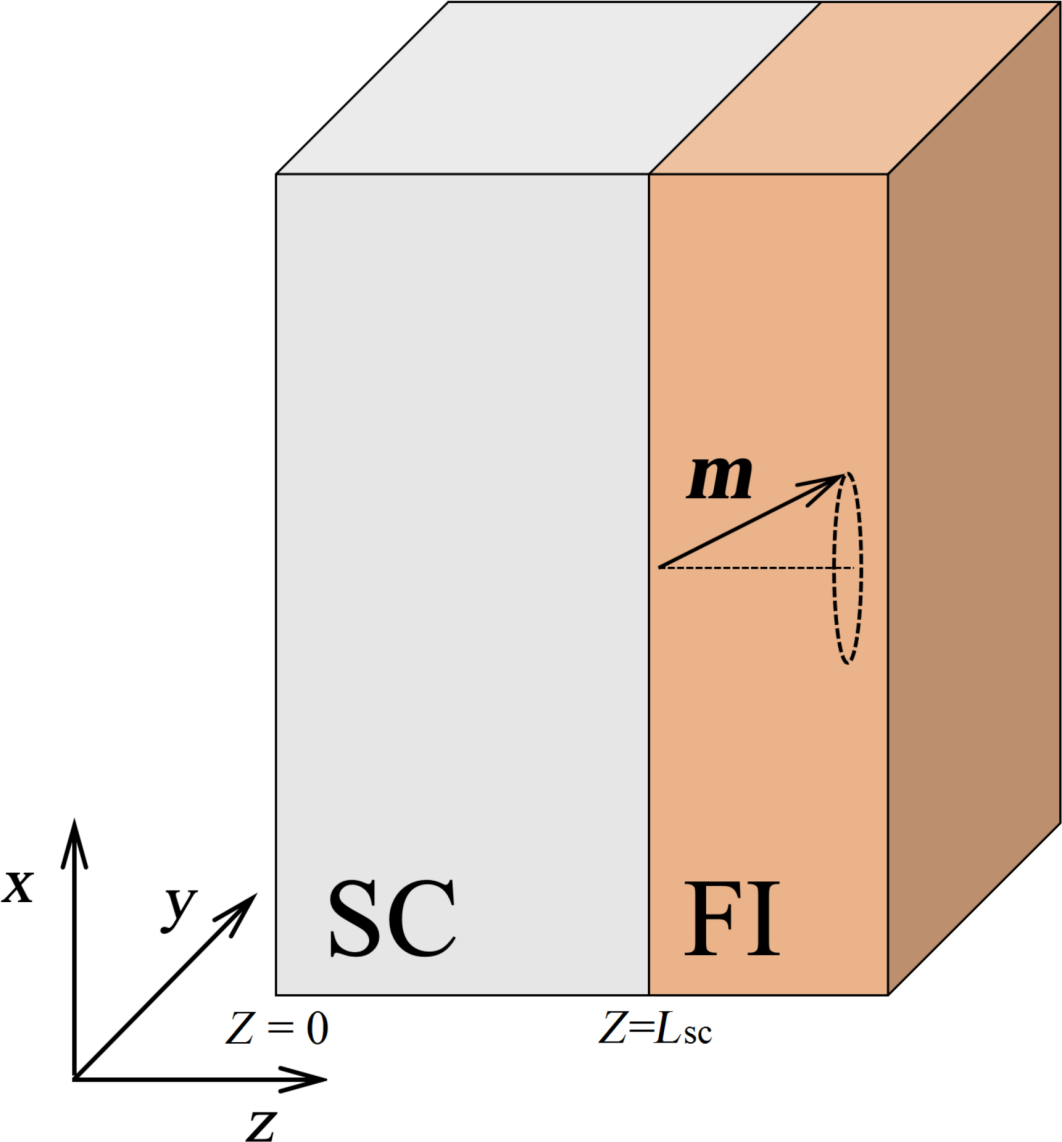
The investigated hybrid superconducting structure consisting of a ferromagnetic insulator (FI) adjacent to a superconductor (SC). The interface between the superconducting layer and free space is located at *z* = 0; the interface between the superconducting layer and the ferromagnetic insulator is located at *z* = *L*_sc_. The magnetization **m** in the ferromagnetic insulator layer is uniform and precesses with the cyclic frequency Ω.

The investigated structure is schematically presented in Figure 1. The spin current is injected from the ferromagnetic insulator (FI) to the superconducting film (SC). The thickness of the ferromagnetic insulator does not matter, because the superconducting correlations do not penetrate into the insulating material. Uniform magnetization periodically precesses in the ferromagnetic insulator with a cyclic frequency Ω. To describe the nonstationary state of the superconducting condensate, we use the formalism of two-time quasiclassical Green’s functions in Nambu–spin-Keldysh space [28]. We expand the Green’s function assuming a weak proximity effect [28] with the ferromagnetic insulator: 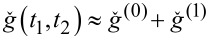. To handle the expansion of the order parameter correctly, we should cancel the odd orders of the perturbation, because the triplet Green’s function components do not contribute to the order parameter. Only even orders of the perturbation series determine its correction. Thus, the superconducting order parameter in the linear regime, has only a zero-order term in expansion.

The resulting dynamics of the superconducting condensate in the weak proximity effect regime can be described via the nonstationary Usadel equation [18–19,30]:


[1]

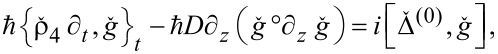



where 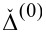 is the stationary BCS superconducting order parameter matrix [28], *D* is the diffusion constant, 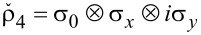 is the auxiliary matrix in Nambu–spin-Keldysh space, 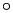 is the time convolution operator, and the anticommutator {*f*, *g*}*_t_* = *f*(*t*_1_)*g*(*t*_1_, *t*_2_) + *g*(*t*_1_, *t*_2_)*f*(*t*_2_). We have dropped the coordinate dependence of the Green’s functions for simplicity of notation. We consider the time-dependent magnetization at the interface as an adiabatic perturbation that changes slowly compared to the timescale of the superconducting system: ℏΩ ≪ Δ. A similar approach was used in our recent work [29].

In general, the equation can be solved numerically within the mixed representation formalism [31]. Sometimes, the Usadel equation (Equation 1) can be solved analytically, for example, in the case of weak superconductivity, as it has been done in the pioneering work by Houzet [32]. Two-time quasiclassical Green’s functions have the following structure [28]:


[2]

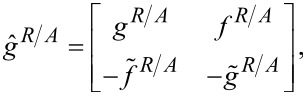




[3]

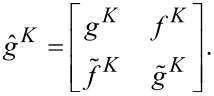



The time-periodicity condition allows for the representation of spin current and induced magnetization as time-harmonic variables:


[4] $$j_{z}^{s}\left(z,t\right)=j_{z}^{s}\left(z\right)e^{i\Omega t},$$

[5] $$M\left(z,t\right)=M\left(z\right)e^{i\Omega t},$$

where *t* = (*t*_1_ + *t*_2_)/2 is the center-of-mass time argument. To form a closed set of equations, we should add the equation for the normalization condition [30] in mixed representation. The amplitudes of the observables in Equation 4 and Equation 5 can be calculated from the Fourier components of quasiclassical Green’s functions using a standard procedure [29]:


[6]

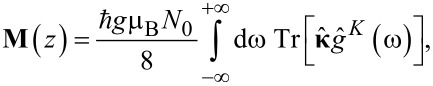



where 
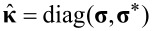
 is the spin operator in electron–hole–spin space, *g* is the gyromagnetic ratio for free electrons, μ_B_ is the Bohr magneton, *N*_0_ is the density of states at the Fermi level, and 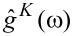 is the Fourier–Winger transform of the Green’s function [29–30] The expression for the spin current takes the following form [29]:


[7]

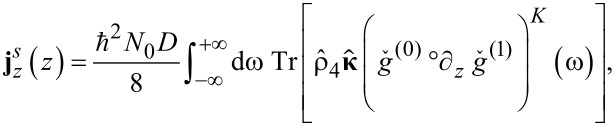



where *K* is the Keldysh component.

Using the normalization condition, Keldysh Green’s function can be written through the distribution function 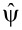: 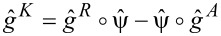. In thermal equilibrium, the distribution matrix reduces to a function tanh(βℏω/2), β = 1/*kT*, which corresponds to the Fermi distribution function.

## Results and Discussion

For the numerical calculations, we have considered niobium as a superconducting metal with the following parameters: *T*_c_ = 9.2 K, Δ*^(0)^* ≈ 1.76*k*_B_·*T*_c_ = 1.4 meV, *D* = 0.8·10^−3^ m^2^·s^−1^, and ε_F_ ≈ 5.32 eV. We approximate the DOS on the Fermi level with the free electron gas value *N*_0_ ≈ 4.9·10^46^ J^−1^·m^−3^. The coherence length has been estimated using 
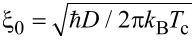
, where *k*_B_ is the Boltzmann constant and ξ_0_ ≈ 11 nm. We numerically solve Equation 1 in mixed representation with the normalization condition. To obtain physical observables from the quasiclassical Green’s functions, one should find the harmonic coefficients in Equation 6 and Equation 7 and directly calculate observable values at the space-time points. In this work, we are interested in the calculation of spin current distributions along the thickness of the superconducting film, as well as the influence of induced magnetization dynamics on the electron perturbations in the S film. The dynamics of any observable will be periodic and can be characterized by its amplitude value. Thus, we only need to calculate the doubled absolute value of the coefficients in Equation 6 and Equation 7, which are exactly the amplitudes of the spin current and magnetization in the linear regime. Nonadiabatic processes are unlikely because the ratio Δ/ℏΩ ≫ 1 for the Nb/Y_3_Fe_5_O_12_ (YIG) hybrid structure. However, the superconducting order parameter may be partially reduced near the S/FI interface because of the inverse proximity effect. It gives rise to the spin distribution of quasiparticles with energies close to the spectrum gap near the interface.

Both spin current and induced magnetization in the superconductor originate from the singlet–triplet Cooper pair conversion mechanism, which is the main origin of the inverse proximity effect. The spin current can be induced only by the nonstationary flow of triplet Cooper pairs, just as in a conventional spin-pumping bilayer structure with a normal metal [33]. Thus, spin currents cannot emerge when the magnetization is stationary inside the ferromagnetic insulator layer. However, there is a possibility to induce stationary pure spin currents inside trilayer superconducting structures [1].

**Figure 2:**
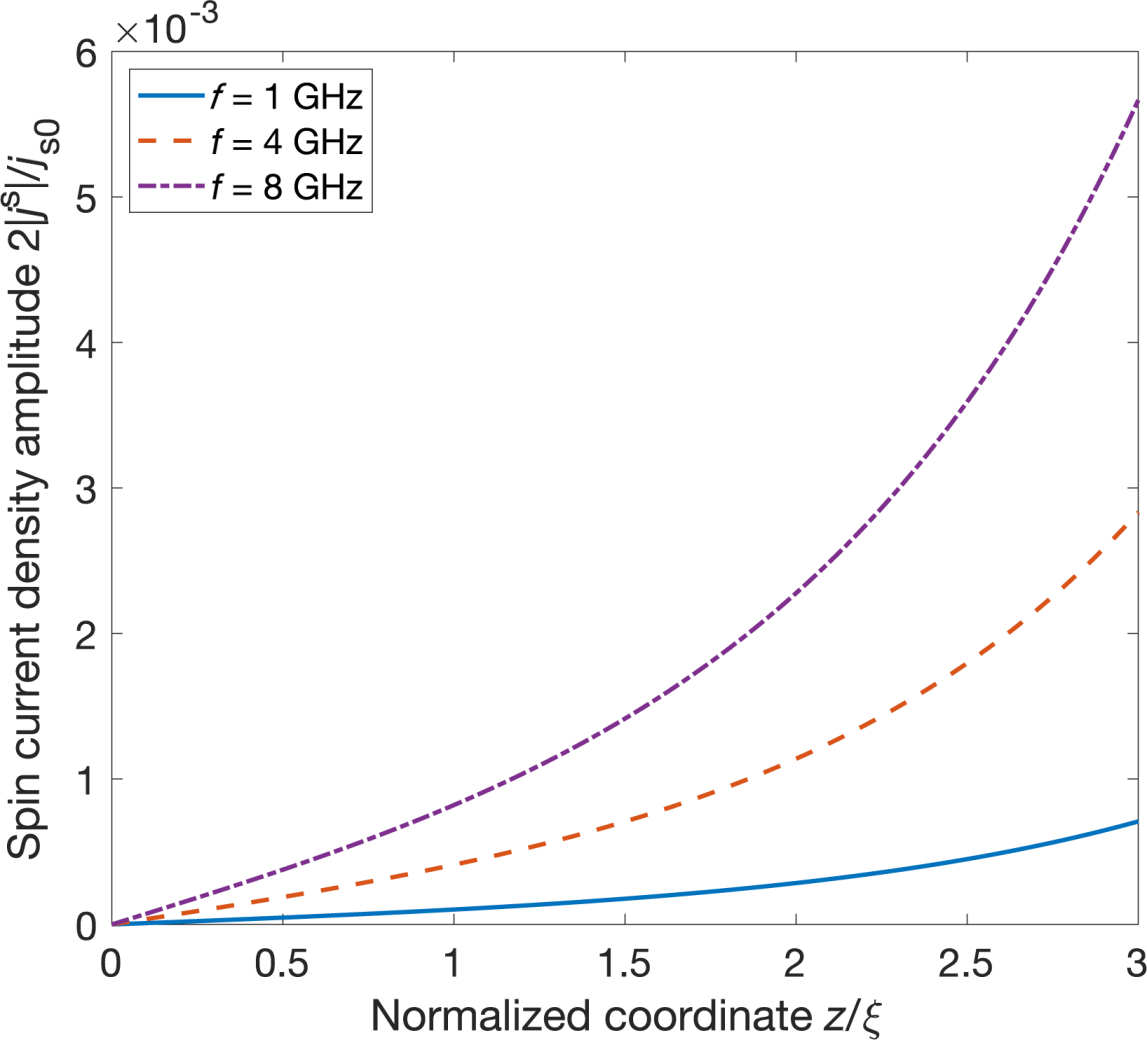
Distributions of the spin current density inside the superconducting layer at different frequencies of the magnetization precession. The interface between the superconducting layer and ferromagnetic insulator is located at *z* = 3ξ

The distributions of spin current amplitudes into the S layer are depicted in Figure 2. The amplitudes are normalized by the factor *j_s0_* = (ℏ/2*e*)*j_e0_*. The charge current density normalization factor is *j_e0_* = 2*eN*_0_*D*Δ*^(0)^*/ξ = 6.262·10^6^ A·cm^−2^.

Distributions of spin current and induced magnetization for aluminum were calculated in our previous work [29]. One can see that the spin current amplitudes decay at a distance of the coherence length, similarly to the induced magnetization. However, the amplitude of the spin current strongly depends on the frequency of the magnetization precession. This effect is similar to ferromagnetic resonance spin pumping in normal metal/ferromagnetic insulator structures. In the last case (normal metal), the decay of the spin current is a consequence of spin relaxation processes, but we do not take into account any spin relaxation mechanisms within our model for a superconductor. We should mention that both spin pumping mechanisms in superconductors and normal metals are determined by the penetration of nonequillibrium spin density from the interface. In metals, such a penetration is limited by the spin flip scattering, while inside the superconductor, the spin relaxation time is usually much longer. Thus, induced magnetization and spin current in our problem are determined mainly by the competition between spin singlet and spin triplet orders [34] Therefore, we conclude that the main mechanism of the spin current decay is similar to that for the induced magnetization. It corresponds to the lowering of the triplet pair density away from the magnetic interface where the singlet–triplet conversion occurs. Moreover, we should point out that the decrease of spin current inside the superconducting layer completely agrees with the boundary condition of the zero matrix current at the interface between the free space and superconducting layer at *z* = 0.

Now let us consider the Fourier coefficients for the induced magnetization. Earlier, we have shown that the induced magnetization almost does not depend on the precession frequency [29]. This is because the absolute value of the projection of the magnetization vector to the interface plane does not change with a change of the precession frequency and may be given by the stationary component of the induced magnetization [35].

**Figure 3:**
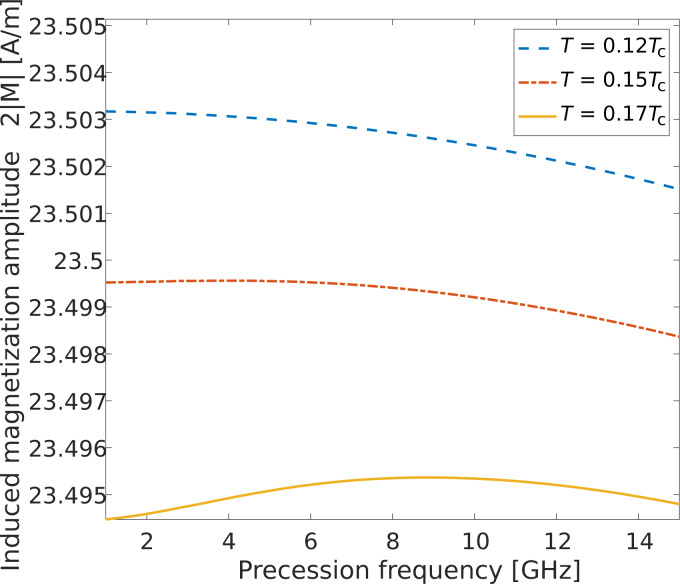
In-plane component of the induced magnetization at the S/FI interface as a function of the magnetic precession frequency at different temperatures.

However, more precise results presented in Figure 3 show that the induced magnetization at the interface depends nonmonotonically on the precession frequency. Moreover, a maximum becomes obvious with increasing temperature, even if we do not take into account the thermal suppression of the superconducting order parameter. The competition between two different spin pumping mechanisms can explain this interesting behavior. The first mechanism is the adiabatic spin pumping of the superconducting condensate, and the second one is the spin pumping of the thermally generated quasiparticles, for example, unpaired electrons and holes. The competition of these two spin pumping mechanisms gives rise to the nonmonotonous frequency dependence of the induced magnetization, which is the sum of the quasiparticle spin density and the triplet correlations component.

**Figure 4:**
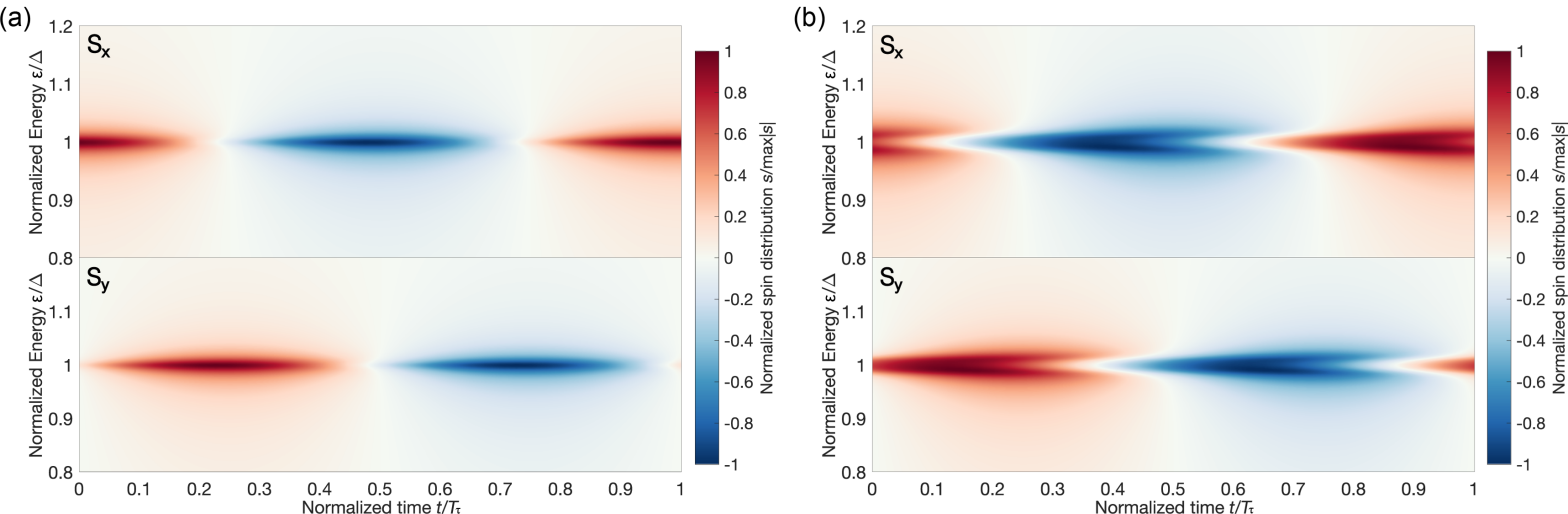
Evolution of the spin-resolved distribution function at the S/FI interface (*S_x_* component in the upper panels and *S_y_* component in the lower panels) at magnetization precession frequencies of (a) 1 GHz and (b) 8 GHz. The normalized time is equal to *t*/*T*_τ_, where *T*_τ_ = 2π/Ω is the period of magnetization precession.

**Figure 5:**
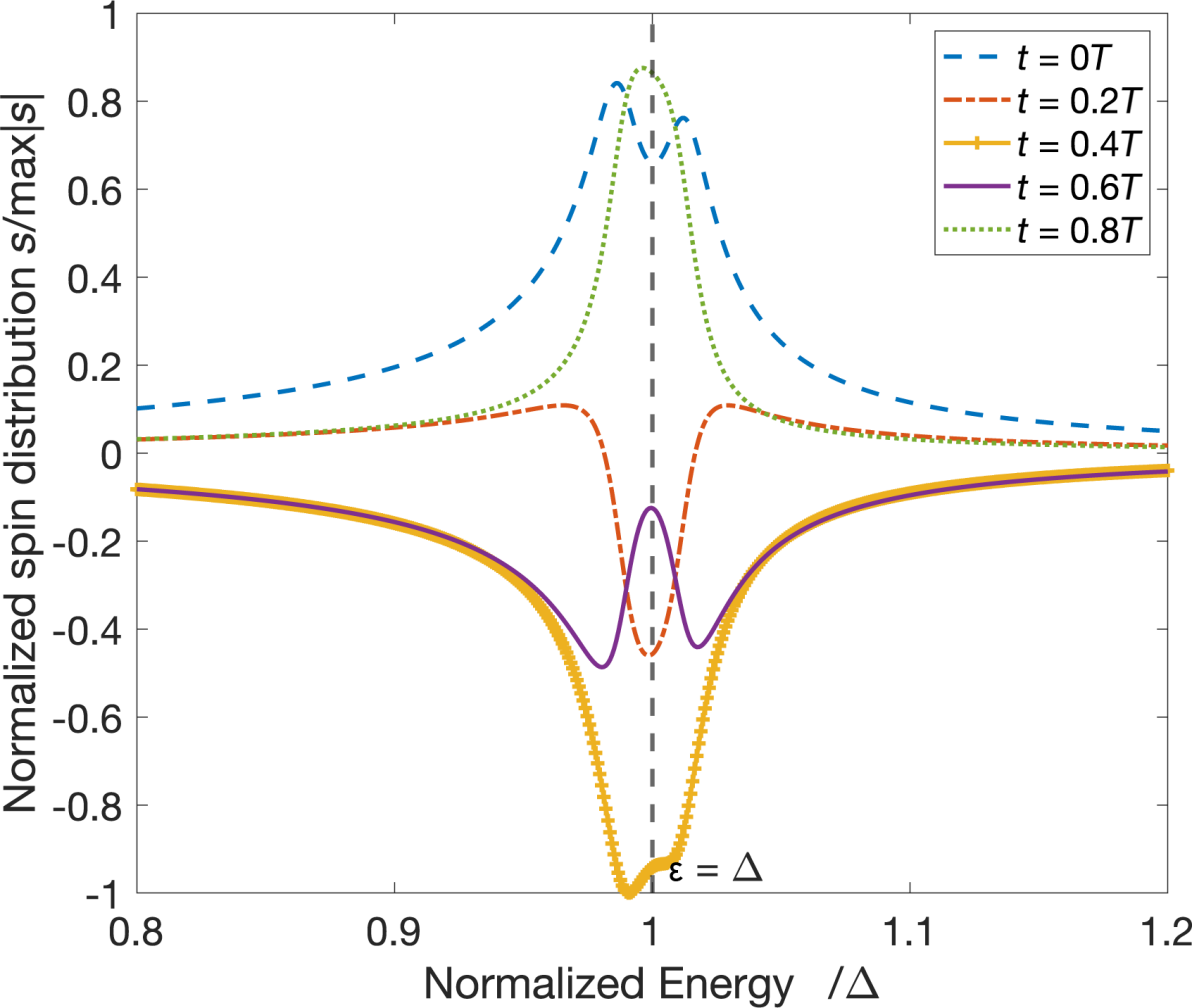
Snapshots of the spin distribution function at different moments of the precession period at a frequency of 8 GHz.

The interplay between magnetization precession and proximity effect can suppress superconductivity at the interface causing an increasing number of quasiparticles. To explore the spin dynamics of the quasiparticles more deeply, let us investigate spin components of the electron block of the distribution function. Spin polarization of quasiparticles can be obtained by applying a spin polarization operator to the distribution function matrix. Due to the block-diagonal structure of the spin operator in electron–hole space, the spin distribution of quasiparticles can be represented as a superposition of electron-like and hole-like spin distributions 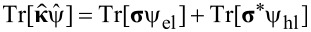. The first term in this expression corresponds to the spin polarization of electron-like quasiparticles and is mathematically equivalent to the trace of the product of Pauli matrix and the left upper block of the distribution matrix. Figure 4 illustrates the dynamics of the quasiparticle distribution function at magnetization precession frequencies of 1 and 8 GHz. The color maps for quasiparticles with *x* and *y* spin component evolution *S_x,y_*(*z*, ε, *t*) = Tr[σ*_x,y_*ψ_el_] are presented in Figure 4. The spin distribution function splits into two almost symmetric peaks around the spectrum gap value with increasing frequency (Figure 5). The asymmetry of the electron spin distribution is very small but visible in Figure 5, where two peaks emerge twice during one period of magnetization oscillation. This picture is similar for hole excitations due to the electron–hole symmetry. It should be noticed that a fraction of the spin distribution is lying inside the gap and should not be taken into account. But in the time-dependent case, there is always an energy shift equal to ±ℏ*Ω/2*. This energy shift appears in every time convolution. The real consequences of these undergap states may be found if one takes into account also the density of states correction, which is beyond the scope of the current paper. Thus, the effect of spin distribution function splitting can be revealed in superconducting hybrid systems with nonequilibrium electron–hole distributions such as superconductor/normal metal contacts [36]. This is one more evidence of the significant role of quasiparticles in the spin dynamics of hybrid superconducting structures.

## Conclusion

In this work, we have investigated the simplest case of the linear adiabatic dynamics of spin current and spin polarization of quasiparticles caused by the proximity of a superconductor to a ferromagnetic insulator. It was found that the spin current density amplitude is proportional to the frequency of the magnetization precession. Spin supercurrent distributions are similar to those of the spin pumping in normal metal/ferromagnetic insulator hybrid structures. But the spin current penetrates into the superconducting film to distances much longer than in normal metals. This behavior is a result of the adiabatic singlet–triplet Cooper pair conversion process at the interface, that is, the inverse proximity effect. We have found that the induced magnetization at the interface has a weakly nonmonotonous dependence on the magnetization precession frequency with a maximum appearing at increasing temperatures. We suppose that this effect originates from spin pumping of quasiparticles, which can be generated at the interface. There is also a dynamical effect of spin splitting of the quasiparticle distribution. This effect can have some nontrivial consequences in superconducting systems with broken electron–hole symmetry. All these effects emerge in the adiabatic regime. The results demonstrate the rich potential of the dynamic inverse proximity effect in hybrid superconductor/ferromagnetic insulator structures, making them promising candidates for novel spintronic devices.
